# Market strategies used by processed food manufacturers to increase and consolidate their power: a systematic review and document analysis

**DOI:** 10.1186/s12992-021-00667-7

**Published:** 2021-01-26

**Authors:** Benjamin Wood, Owain Williams, Vijaya Nagarajan, Gary Sacks

**Affiliations:** 1 Global Obesity Centre, Deakin University, Melbourne, Australia; 2 School of Political Science and International Studies, University of Leeds, Leeds, UK; 3 Macquarie Law School, Macquarie University, Sydney, Australia

**Keywords:** Market strategy, Processed food manufacturers, Market power, Commercial determinants of health, Food systems

## Abstract

**Background:**

The public health community has become increasingly critical of the role that powerful corporations play in driving unhealthy diets, one of the leading contributors to the global burden of disease. While a substantial amount of work has examined the political strategies used by dominant processed food manufacturers that undermine public health, less attention has been paid to their use of market strategies to build and consolidate power. In this light, this paper aimed to systematically review and synthesise the market strategies deployed by dominant processed food manufacturers to increase and consolidate their power.

**Methods:**

A systematic review and document analysis of public health, business, legal and media content databases (Scopus, Medline, ABI Inform, Business Source Complete, Thomas Reuters Westlaw, Lexis Advance, Factiva, NewsBank), and grey literature were conducted. Data extracted were analysed thematically using an approach informed by Porter’s ‘Five Forces’ framework.

**Results:**

213 documents met inclusion criteria. The market strategies (*n*=21) and related practices of dominant processed food manufacturers identified in the documents were categorised into a typological framework consisting of six interconnected strategic objectives: i) reduce intense competition with equivalent sized rivals and maintaining dominance over smaller rivals; ii) raise barriers to market entry by new competitors; iii) counter the threat of market disruptors and drive dietary displacement in favour of their products; iv) increase firm buyer power over suppliers; v) increase firm seller power over retailers and distributors; and vi) leverage informational power asymmetries in relations with consumers.

**Conclusions:**

The typological framework is well-placed to inform general and jurisdiction-specific market strategy analyses of dominant processed food manufacturers, and has the potential to assist in identifying countervailing public policies, such as those related to merger control, unfair trading practices, and public procurement, that could be used to address market-power imbalances as part of efforts to improve population diets.

**Supplementary Information:**

The online version contains supplementary material available at 10.1186/s12992-021-00667-7.

## Background

Unhealthy diets are one of the leading contributors to the global burden of disease, accounting for an estimated 11 million deaths each year and approximately 15% of all years lost to ill-health [1]. The public health community has increasingly become critical of the role of powerful private actors, such as transnational food manufacturers, in driving unhealthy diets through the shaping of food supply chains, food environments and consumer behaviour [2–5]. To date, the main focus of such work, including as part of the emerging field of the commercial determinants of health (CDoH), has examined: the links between unhealthy food products and adverse population health outcomes; the underlying global drivers and institutional arrangements that promote corporate interests and transnationalise their activities; and the use of corporate political strategies, such as political donations, lobbying, and regulatory capture, by dominant processed food manufacturers that undermine public health policy and practice [6–10].

To date, however, the market strategies deployed by processed food manufacturers have received limited attention from the public health community. Most public health research into the market strategies of food companies has focused on the public health implications of marketing, product reformulation, and food labelling [11–18]. However, large firms are known to deploy a wide range of strategies to protect their business models and products from adverse regulation, while simultaneously building and preserving market dominance and profits [19–21]. In order to understand the ways in which companies in the food industry act to maximise profits and power, it is critical that the public health community has the tools to monitor and analyse the full range of strategies used.

Drawing on strategic management literature, ‘market strategies’ can be defined as concerted patterns of actions taken in the market environment for the purpose of improving corporate performance (i.e. maximising profits and shareholder returns), for example, in actions such as pricing or merger and acquisitions [19]. From the perspective of Michael Porter, who is often portrayed as the founder of modern market strategy, effective deployment of market strategies requires an understanding of the competitive structure of the industry in question [19, 22]. For example, in an industry characterised by intense rivalry, a firm unable to compete on price may choose to instead target a new consumer segment of the market by adapting the features of its product or service to the target demographic. As another example, an incumbent firm may decide to acquire new firms that enter the market to reduce the risk of losing market share [22]. Effectively, a firm’s choice of appropriate market strategies, according to Porter, involves assessing how weaknesses in the competitiveness of an industry can be leveraged in order to generate profits [21]. Such a perspective highlights the links between the deployment of effective market strategies and the potential need for government intervention to mitigate or prevent the use of strategies that restrict competition [23].

Importantly, market strategies operate in tandem with non-market strategies. Non-market strategies include a cluster of practices that are designed to improve overall corporate performance by influencing the interconnected policy, regulatory, institutional, ideological and broader socio-political structures that shape market environments [19, 24]. In many cases, the distinction between market and non-market strategies can be artificial, as both are often collapsed into single actions or activities undertaken by firms. For example, corporate social responsibility strategies are often perceived as having both a market-strategy dimension (e.g. by increasing brand value) and a non-market strategy dimension (e.g. through gaining political and consumer legitimacy) [25]. As such, analysis of market strategies often cannot be divorced from analysis of non-market strategies, as they can be mutually interdependent and overlapping [20]. Nevertheless, considering market strategies independently and exclusively is valuable and important as a heuristic device, especially for understanding how market-based power imbalances are created or exacerbated, and, more broadly, for understanding corporate power and behaviours [26].

In this paper, we focus on the market strategies used by processed food and beverage manufacturers with concentrated and substantial market power (hereinafter dominant food companies). Many of these dominant food companies are transnational in nature, at least from a production and consumption perspective. However, there are several cases where particular companies are dominant in only one national market. In addition, it is important to highlight that markets have geographical boundaries, and regulatory agencies typically interpret market power as being confined within these boundaries [27]. The majority of dominant food companies predominately manufacture and distribute branded processed food products [3, 28], defined, for the purposes of this paper, as firms that process post-harvest agricultural commodities (as well as intermediate inputs from primary processing firms and/or synthetic food ingredient manufacturers) into processed foodstuffs [29]. We make a distinction between primary and secondary processors. Primary processors typically add value by processing raw agricultural commodities, such as refining grains, crushing oilseeds or slaughtering animals for meat packing [30]. Examples of prominent firms that undertake primary processing activities include JBS, Archer Daniels Midland, and Bunge. In comparison, secondary processors primarily add value to processed ingredients, such as corn syrup or refined sugar, by manufacturing highly processed foods [30]. Examples of prominent firms that undertake secondary processing activities include The Coca-Cola Company, the world’s largest soft drink manufacturer, and Nestle, the world’s largest food manufacturer that operates across a number of different processed food markets, including confectionery, ice cream, and processed dairy products [31, 32]. It is important to note that many large firms operate across functional levels in the food value chain, and many food manufacturers undertake both primary and secondary food processing activities.

In many food industries across the world, the processed food manufacturing sector is highly oligopolistic in terms of the market structure (i.e., dominated by only a few firms) and characterisable by a large number of suppliers that are only able to sell their products to a small number of dominant food companies (i.e. these constituting an oligopsony) [33]. Additionally, processed foods markets typically have substantial barriers to market entry that further consolidate the market power of incumbent firms [33].

**Fig. 1:**
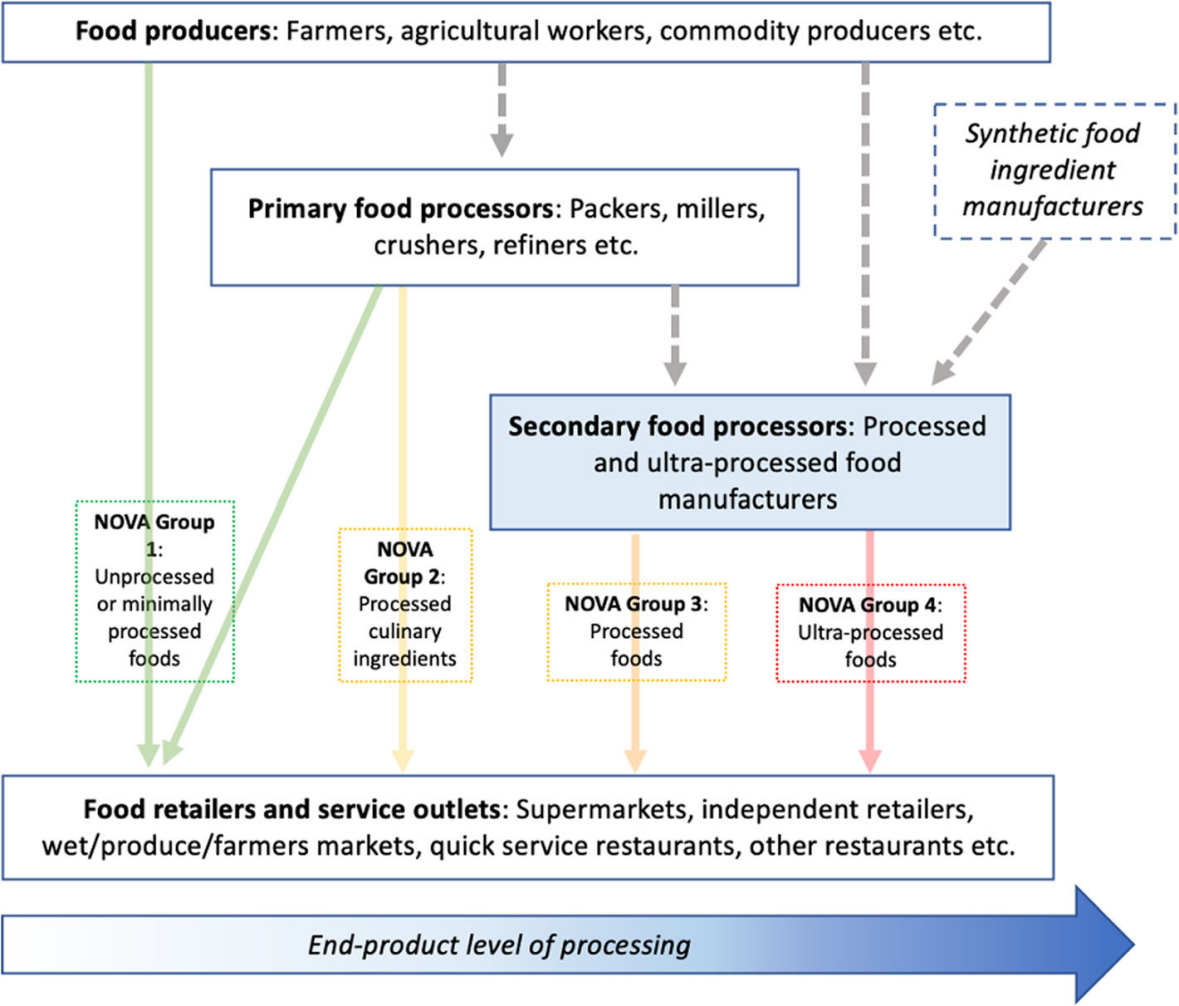
A schematic conceptualisation of different pathways in the food value chain based on the level of processing of end-products

The concentrated market power of dominant food companies is a public health concern because it confers them with the ability to promote and reinforce food value chains and food environments geared towards the production and consumption of their products, many of which are highly processed. We draw from the food classification system NOVA to support the rationale for focusing on dominant processed food companies (*see* Fig. 1) [34]. The NOVA system, an increasingly used food classification system in public health nutrition-related research, categorises food products into four groups according to their level of processing [35, 36]. The first group is unprocessed and minimally processed foods. The second group is processed culinary ingredients, which includes oils, butter, sugar, lard, and salt. The third group is processed foods, which are made by adding salt, oil, sugar or other substances from the second group to foods from the first group. The fourth and final group are ultra-processed foods (UPFs), which include soft drinks, confectionery, sweet biscuits, ice-cream, and savoury snacks [37, 38]. UPFs are made from combining substances derived from foods with synthetic additives, typically via a series of industrial techniques and processes. Importantly, a key feature of UPFs is that they contain food substances that are never, or at most very rarely, used in domestic kitchens, meaning that they are almost exclusively made by processed food manufacturers [34]. UPFs now amount to around half of the total dietary energy consumed in highly developed market economies, and their sales in less developed market economies are increasing rapidly [34]. Studies have shown that a greater contribution of UPFs to total energy intake are associated with poorer diet quality [39–41], higher risks of all-cause mortality [42, 43], obesity [44, 45], and a range of diet-related chronic diseases, including depression [46–52].

A second important public health implication of the well-recognised concentrated market power of dominant food companies is that firms use their market power to generate profits in excess of what could occur in competitive markets. This accumulation of excessive material resources can, in turn, be used to fund corporate strategies and practices that undermine public health, such as lobbying, intense marketing, public relations, and the funding of scientific research and institutions [2–4, 53, 54].

In light of the current gaps in the public health literature, the aim of this review was to systematically review the market strategies deployed by dominant processed food manufacturers to build and consolidate their power, and to develop a typological market strategy framework to inform comprehensive analysis of the market strategies and practices used by dominant processed food manufacturers from a public health perspective.

## Methods

This review was undertaken in accordance with the Preferred Reporting Items for Systematic Reviews and Meta-Analyses (PRISMA) statement [55]. Original studies, reviews, commentaries, editorials, books and book chapters, working papers, online news articles, court case summaries, market research reports, and web documents from intergovernmental and non-governmental organisations were included if they met the inclusion criteria.

### Search strategy

The search strategy was developed with the assistance of a specialist librarian. Preliminary search testing was conducted to find keyword search terms that captured the market strategies used by dominant food companies to exercise or consolidate market power (*refer to supplementary file for detailed search strings*). Public health, social sciences, and business and strategic management literature were searched via Scopus, Web of Science, Medline, Business Source Complete, and ABI Inform databases. Competition law and policy literature was searched using Thomas Reuters Westlaw and Lexis Advance. We used Factiva and NewsBank to search online news articles. A grey literature search was conducted using Google Scholar, Google Advanced Search, the market research databases Euromonitor International (Passport) and IBIS World, and a targeted search of the websites of the following intergovernmental and non-governmental organisations: the Food and Agricultural Organization of the United Nations (FAO), the Organization for Economic Cooperation and Development (OECD), the United Nations Conference on Trade and Development (UNCTAD), the International Panel of Experts on Sustainable Food Systems (IPES-Food), and ETC Group. Finally, backward snowballing was applied in which relevant references of the identified documents were also reviewed. Databases were searched on 7 July 2020, with all results exported and duplicates removed. Grey literature searches were conducted between 8 and 10 July 2020. The first 10 pages of Google Scholar and Google were screened. Citation searches were conducted between July and September 2020.

### Literature selection

The review attempted to identify documents that described or discussed the use of market strategies and practices by dominant food companies. Titles, abstracts, executive summaries and tables of contents (where relevant) were screened for all search results. Documents were included if they focused on market-based strategies used by dominant food companies, with discussion of the use of market strategies as a means of exercising power vis-à-vis other market-based actors, e.g., other food manufacturers, firms at different functional levels of the food value chain, and consumers. Documents were excluded if they were not published in English. Following screening, available full texts were retrieved, reviewed and tabulated by one of the authors.

### Quality assessment

Given the diversity and multi-disciplinary nature of the data, we considered the application of standard risk of bias assessment tools, e.g. the Cochrane Handbook for Systematic Reviews [56], to be inappropriate. Instead, quality assessment was performed by categorising the sources of data into two different groups. The first group was data that were collected from peer-reviewed articles, court case proceedings and summaries, and authoritative reports from inter-governmental organisations. The second group was data sourced from all other documents, e.g., online media content, market research reports, and working papers. We considered the first group to be of higher quality given that the documents were much less likely to be subject to author bias relative to the second group.

### Data extraction, coding and synthesis

Data extraction was undertaken by the lead author. Data extracted from each text included author(s), date, title, and the discussed market-based activity (or activities) undertaken by the dominant food company (or companies). Generalised discussions of market-based activities of dominant companies active in the food manufacturing sector were also included. The market-based activities were synthesised and categorised into practices (i.e., a specific type of activity) and strategies (i.e., a concerted set of practices deployed to attain a goal).

To inform the process of categorising the consolidated strategies and related practices, we initially developed a list of strategic objectives based on Porter’s ‘Five Competitive Forces’ framework (hereinafter the Five Forces framework). The Five Forces framework is one of the most well-known strategic management and business frameworks [21, 22]. From a market strategy perspective, the Five Forces framework outlines how firms can select and use market strategies based on identifying and leveraging the power imbalances that exist within the market environment [23]. The five forces and their related strategic implications can be described as follows [21, 22]: i) *reduce competitive rivalry* through the use of strategies to reduce intense competition, especially with large rivals, and to maintain dominance over smaller rivals; ii) *counter threat of market entrants* by raising barriers to market entry to make it harder for new firms to enter the market; iii) *counter threat of substitutes* by deploying strategies that protect the firm from having their buyers switch to substitute products; iv) *increase seller power* by consolidating power over buyers; and v) *increase buyer power* by consolidating power over suppliers. We adapted the Five Forces framework for application to the food manufacturing sector by considering ‘substitutes’ to be substitute food products (regardless of their level of processing), ‘suppliers’ to be firms from upstream functional levels (e.g., food production, primary processing), and ‘buyers’ to be both downstream firms (e.g., retailers, food service outlets) and consumers. In addition, we separated the strategic objective to *‘increase seller power’* into two to take into account the differences from a public health perspective between the deployment of strategies used by dominant food companies vis-à-vis retailers and distributors compared to consumers.

The process of consolidating strategies and related practices, and categorising based on the abovementioned strategic objectives, was conducted in an iterative manner. The initial categorisations were reviewed by all of the authors, with discrepancies resolved via a consultative process. In undertaking the categorisations, we recognised that, in some cases, dominant food companies may use the same practice as part of multiple strategies, and, due to the dynamic nature of markets, the list of practices categorised under each strategy is unlikely to be exhaustive. Accordingly, the practices identified are best considered as ‘illustrative’.

Summarised material was presented as a novel typological market strategy framework to inform analysis of the market strategies and related practices used by dominant processed food manufacturers to consolidate power from a public health perspective.

## Results

Database searching returned 1115 results. After grey literature and snowball searching, 213 documents were included for final review (*see supplementary file for PRIMSA flow chart*). In the evidence group considered to be of higher quality, documents included peer-reviewed articles (*n*=67), legal case summaries (*n*=24), and authoritative intergovernmental reports (*n*=12). In the evidence group deemed to be of lower quality, documents included online media content (*n*=60), market, industry or investor research reports (*n*=22), conference papers and manuscripts (*n*=15), non-authoritative reports (*n*=10), completed doctoral theses (*n*=3), and one transcribed speech.

Identified market strategies (*n*=21) were categorised according to six interconnected strategic objectives specific to dominant processed food manufacturers: i) reduce intense competition with equivalent sized rivals and maintain dominance over smaller rivals; ii) raise barriers to market entry by new competitors; iii) counter the threat of market disruptors and drive dietary displacement in favour of their products; iv) increase firm buyer power over upstream food supply chain actors; v) increase firm selling power over downstream food supply chain actors, and vi) leverage informational power asymmetries in relations with consumers *(see* Table 1*)*.

**Table 1:** A typological framework of the strategic objectives and associated market strategies and related practices used by dominant processed food manufacturers to increase and consolidate market power

Strategic Objective	Strategy	Illustrative practices	Evidence (by group)
**Reduce intense competition with equivalent sized rivals and maintain dominance over smaller rivals**	Horizontal integration	Acquisition of rival in the same product and geographic market	Group I: [29, 57–70] Group II: [71–82]
	Direct transnational expansion	Direct investment in foreign markets through acquisition of foreign food manufacturing firms	Group I: [2, 3, 29, 63, 64, 83–85] Group II: [71, 73, 74, 82, 86–92]
		Substantial investments in locally acquired firms to penetrate emerging markets via boosting production and marketing capabilities	Group I: [2] Group II: [79, 88]
	Horizontal collusion	Explicitly collude by entering price and/or output fixing arrangements with rivals	Group I: [63, 93–99] Group II: [100–106]
		Tacitly collude with rivals by coordinating behaviour	Group I: [33, 96, 107]Group II: [106]
	Horizontal collaboration	Risk-spreading arrangements with rivals to penetrate product markets	Group I: [29, 108] Group II: [61, 82, 109–115]
		Risk-spreading arrangements with local manufacturing firms in foreign markets to penetrate new geographic markets	Group I: [116] Group II: [74, 79, 87, 114, 117]
	Anti-competitive pricing strategies	Exploit dominant market position to increase prices and profit margins	Group I: [107, 118]
		Anti-competitive pricing practices to push competitors out of markets, e.g. predatory pricing and territorial price discrimination	Group I: [119–124] Group II: [125]
	Create, maintain and increase consumer demand	Invest in intense and aggressive marketing practices to create/maintain/increase consumer demand for branded products in new and existing markets	Group I: [2, 3, 11, 84, 85, 116, 126–129] Group II: [91, 130–133]
		Segment consumer markets through marketing and price segmentation practices	Group I: [15, 64, 83, 84, 116, 134, 135] Group II: [109, 132, 133, 136–140]
**Raise barriers to market entry by new competitors**	Develop, acquire and protect value of brands and other intangible assets	Make substantial investments in marketing practices to increase and protect the equity of owned brands and brand loyalty	Group I: [29, 58, 63, 64, 118, 141, 142] Group II: [82, 132, 143–152]
		Build brand equity and loyalty by differentiating products based on different qualities and characteristics	Group I: [33, 64, 107, 134, 153–156] Group II: [81, 109, 113, 117, 130, 132, 138, 140, 146, 147, 157–160]
		Increase value of owned intangible assets via acquisition of intangible assets of other firms, and make substantial investments in product and process innovation	Group I: [65, 66, 161, 162] Group II: [163, 164]
		Protect intangible assets, including brands, through intellectual property right channels whenever possible	Group I: [64, 165] Group II: [91, 166–169]
	Exploit economies of scale (production, marketing and financial)	Increase productive efficiency and minimise marginal cost of production through practices that achieve production economies of scale	Group I: [33, 58, 64, 119, 161, 162] Group II: [79, 88, 132, 147, 149, 150, 163, 170]
		Exploit marketing economies of scale by spreading large marketing budgets across a greater range of effective marketing media	Group II: [74]
		Exploit financial economies of scale to make large capital investments and to deploy cost-cutting financial strategies (e.g. transfer pricing)	Group I: [171, 172] Group II: [88, 147, 151, 152, 173]
	Supply chain control	Control distribution channels using trading practices such as exclusive dealing arrangements, predatory foreclosure, category ‘captaincy’, slotting fees, and calendar marketing agreements	Group I: [29, 58, 99, 118, 123, 127, 153, 174–182] Group II: [101, 147, 183–188]
		Establish distribution networks in hard-to-access areas	Group I: [135, 154] Group II: [147]
		Supply diversification and competitive sourcing to secure cheap inputs	Group I: [116, 162, 189, 190] Group II: [82, 132, 191, 192]
		Update and streamline supply management practices (e.g., automation in production processes) to boost productive efficiency, minimise transaction costs and promote product differentiation	Group I: [155, 193–195] Group II: [82, 147, 196–198]
		Control ancillary activities by acquiring firms and related assets in ancillary industries, e.g. business management services, storage and transport industries	Group I: [162, 199] Group II: [200]
		Substantial investments in Big Data platform technologies to acquire, control and manipulate large amounts of supply chain and consumer related information	Group II: [132, 201]
**Counter the threat of market disruptors and drive dietary displacement in favour of their food products**	Product diversification (increase economies of scope)	Acquisition of firms in substitute product markets (domestic or cross-border)	Group I: [29, 57, 58, 63, 107, 156, 171, 202] Group II: [32, 74, 79, 88, 89, 109, 159, 164, 167, 203–206]
		Acquisition of firms in related (e.g. healthcare, pet food) or unrelated industries (conglomerate)	Group I: [107, 108, 207] Group II: [72, 109, 208]
		Substantial investments in the development of new products in response to consumer trends	Group II: [32, 206, 209]
		Enter and penetrate related product markets through strategic alliances, joint ventures and co-branding agreements	Group I: [210] Group II: [74, 109, 113, 211]
	Control market disruptors	Operate as venture capitalists and business incubators in order to eliminate the threat of start-ups and capitalise on and internalise successful start-ups	Group II: [109, 206]
	Drive dietary displacement and adaption in favour of branded processed foods over alternatives	Engineer hyperpalatable, quasi-addictive, and aesthetically and texturally pleasing foods	Group I: [2, 34, 85, 129, 154, 212] Group II: [213, 214]
		Drive changes in food consumption habits, e.g. promotion of snacking over regular meals, that favour the consumption of branded processed foods	Group I: [85]
		Keep price of branded products low in value-based food markets by taking advantage of economies of scale and cheap commodity inputs	Group I: [2, 3, 129] Group II: [91]
**Increase firm buyer power via exercising power over upstream food supply chain actors**	Vertical integration (backwards)	Acquisition of upstream firms and related assets	Group I: [58, 63, 107, 194, 195, 215, 216] Group II: [132, 217]
	Vertical control (backwards)	Control access to and use of inputs, e.g. land, production-related plant and equipment, crop varieties, water	Group I: [29, 33, 58, 64, 141, 195, 218] Group II: [102, 201, 217, 219, 220]
		Use private standards to control upstream firms	Group I: [29, 190, 221, 222] Group II: [132]
		Exert power over suppliers through the use of unfair contract agreements	Group I: [29, 58, 189, 223, 224] Group II: [225, 226]
	Vertical coordination (backwards)	Coordinate upstream activities through risk-spreading arrangements (e.g., strategic alliances and joint ventures) with upstream firms	Group I: [64, 108, 227, 228] Group II: [82, 217, 229, 230]
**Increase firm seller power via exercising power over downstream actors**	Vertical integration (forwards)	Acquisition of downstream firms and related assets	Group I: [107, 153, 195, 215] Group II: [187, 198, 231]
	Vertical control (forwards)	Control distribution channels using practices such as exclusive dealing arrangements, loyalty rebates, product bundling, resale price maintenance, and territorial supply contracts	Group I: [99, 174, 181, 232] Group II: [124]
	Vertical coordination (forwards)	Coordinate downstream processes and activities through risk-spreading arrangements	Group I: [228, 233] Group II: [206]
	Vertical collusion	Collude with retailers to set up price-fixing arrangements of branded products	Group I: [63] Group II: [103]
**Leverage informational power asymmetries in their relations with consumers**	Drive demand within vulnerable consumer groups	Target vulnerable population groups with integrated marketing communication practices and use of multiple marketing channels, especially digital channels	Group I: [11, 13–16, 234]
	Exploit product and process related information asymmetries	Withhold, manipulate or use misleading process and product-related information on food labels via practices such as the use of deceptive health and nutrition claims, misleading marketing claims (e.g. unfinished or irrelevant claims), and greenwashing	Group I: [33, 235–237] Group II: [140, 238]

### Reduce intense competition with equivalent sized rivals and maintain dominance over smaller rivals

The review identified six strategies that dominant food companies employ to reduce intense competition with large rivals and maintain market dominance over smaller rivals. *Horizontal integration*, achieved by acquiring a rival firm in the same product market, is an important strategy that increases a firm’s market share and reduces its number of direct rivals [29, 57–61, 63–82, 239]. Horizontal acquisitions provide a substantial competitive advantage by allowing the buying firm to increase its number of brands, achieve economies of scale and economies of scope, obtain new proprietary technologies and processes, and by indirectly increasing its buyer and seller power [58–60, 240]. In some cases, the transaction value of acquisitions can be extremely large, such as the $63 billion price tag on Heinz’s takeover of Kraft Foods in 2015 [29]. The acquisition of food manufacturing firms in foreign markets is also a key means for dominant food companies to enter and penetrate new markets, especially those embedded in emerging economies [2, 3, 29, 63, 64, 71, 73, 74, 82–84, 86–92]. Dominant food companies have largely entered emerging markets via acquisitions, strategies that are often in response to stagnating sales revenue in their home markets [2, 3, 29, 91, 92, 116]. Following entry, dominant food companies often further penetrate emerging markets by investing substantial amounts of capital in boosting production and marketing capabilities [2, 79, 88, 116].

It was identified that dominant food companies also undertake risk-spreading arrangements, such as strategic alliances, licensing agreements, franchising agreements, and joint ventures, in order to penetrate both new and existing product and geographic markets [61, 74, 79, 82, 86, 87, 109, 110, 112, 114–116]. One noteworthy example is the joint venture Cereal Partners Worldwide, created in 1991 between Nestlé and General Mills, which at the time united the marketing capacity of Nestlé with the production know-how of General Mills to penetrate the global breakfast cereal market (to the detriment of Kellogg) [162]. Moreover, risk-spreading arrangements with local manufacturing firms can allow dominant food companies to enter new geographic markets without the need to make big and often risky investments, while still being able to benefit from local distribution channels, manufacturing capabilities, and market/consumer/regulatory knowledge. Many dominant food companies, as global brand owners, can also decide to use licensing agreements – whereby they permit local firms to manufacture and distribute a branded product within a specified region – to generate revenue without taking the risk of investing in local manufacturing or distribution activities [61, 64, 79, 111, 113].

The review identified cases where dominant food companies have resorted to collusion to reduce intense competition and maximise profits. Despite explicit collusion being illegal in many national legal systems, a number of cases were identified in which dominant food companies were reported to have entered price and/or output fixing arrangements, forming cartels with rivals in the same product market [33, 63, 93–107]. In 2008, a German court found that a number of large branded chocolate manufacturers, including Kraft Foods and Alfred Ritter, had colluded to coordinate a series of price increases – in some cases around 15–25% – in the German market [98]. Explicit collusive practices, such as the sharing of sensitive market information, are not specific to illegal cartels and can be facilitated through legal channels such as trade groups and associations [63, 101]. However, collusion in processed food markets has been shown to often be tacit, rather than explicit. Tacit collusion usually occurs in concentrated markets, such as carbonated soft drink markets, when large rivals that compete over time and across regions repeatedly interact and thus begin to anticipate one and another, allowing them to coordinate behaviour with little or no direct communication [33, 96, 106, 107].

It was revealed that dominant food companies at times use a number of *anti-competitive pricing strategies* as a means of maintaining market dominance over smaller rivals. Food companies in positions of market dominance were shown to exploit their ‘price making’ capabilities in which they set prices without having to make reference to the pricing strategies of rivals [107, 118]. In such cases, dominant firms can choose to set their prices high in order to increase profit margins to levels higher than what would be possible under genuinely competitive conditions. Conversely, dominant firms can also set their prices at very low levels to force out would-be competitors. This practice, known as predatory pricing, occurs when dominant firms set prices at very low levels, at times even below the marginal cost of production, for extended periods of time to drive out smaller rivals lacking the power to absorb consequent losses [119, 121, 124, 125]. Similarly, some dominant food companies were shown to employ a practice called territorial price discrimination, whereby predatory prices employed in certain geographical markets are offset by setting higher prices in other markets where the targeted rivals are not active [120, 122].

Dominant food companies are able to leverage their greater financial, production and market power relative to smaller rivals by making substantial investments in a range of practices that *increase consumer demand for their branded products*. It is important to note that the use of this market strategy also raises barriers to market entry, and as such, links with strategies discussed in the next section (*refer to 3.2. Raise barriers to market entry by new competitors*). Dominant food companies reportedly spend enormous amounts of money on intense and aggressive marketing, including product placements and price promotions. This maintains consumer demand for their branded products in existing markets, as well as creating and increasing demand in new markets [2, 3, 11, 84, 85, 91, 116, 126–130, 132, 133]. Furthermore, relative to smaller food manufacturers, dominant food companies tend to be able to negotiate more advantageous product placement and trade promotion agreements with retailers [127].

Similarly, dominant food companies often have the necessary resources to aggressively pursue market segmentation strategies, especially in relation to developing niche markets [15, 64, 83, 84, 109, 116, 120, 132, 133, 135–140]. This strategy involves segmenting different groups of consumers with differentiated products and targeted marketing based on their demographics (e.g. children), income and economic status (e.g. lower income groups), psychographics (e.g. consumer beliefs, values, motivations, priorities) and behavioural characteristics (e.g. purchasing behaviour). Market segmentation can also have a geographic dimension, for example, as evidenced by dominant food companies investing substantial amounts of money into adapting their global brands to the local tastes and habits of different consumer groups around the world, a process often captured by the term ‘glocalisation’ [82, 83, 116, 137]. Furthermore, market segmentation permits firms to tier or differentiate prices, maximising sales and profits by gearing prices to the different abilities to pay across national markets.

### Raise barriers to market entry by new competitors

The review identified three broad strategies used by dominant food companies to raise barriers to market entry in order to prevent unwanted competition, consolidate market power, and facilitate profit maximisation. The identified strategies were *developing, acquiring and protecting the value of their brands and other intangible assets*; *exploiting production, marketing and financial economies of scale*; and obtaining *supply chain control*.

Dominant food companies often invest substantial amounts of capital in increasing and protecting the value of their brands, often referred to as brand equity [29, 58, 63, 81, 82, 118, 132, 141–143, 146, 148–152]. For some perspective, The Coca-Cola Company’s (TCCC) brand portfolio was valued at US$63.4 billion in 2019 [241]. Building a brand is costly and time consuming, giving incumbent firms with existing brand loyalty an enormous advantage over new market entrants [33, 82, 132]. Brand equity is also crucial for dominant food companies to maintain bargaining power over large supermarkets, given that ‘must stock’ brands are much less likely to displaced by supermarket own brands compared with the lesser known brands of smaller food manufacturers [64, 141, 143–145].

A critical aspect of developing and protecting brand equity is product differentiation [33]. Product differentiation describes a strategy of differentiating a firm’s products from those of existing or potential rivals. This is achieved by differentiating products based on characteristics such as taste, convenience, appearance, production processed, healthfulness, marketing arrangements, and the ecological implications of production and consumption [33, 64, 81, 86, 107, 109, 113, 130, 132, 134, 138, 140, 146, 147, 153–160]. For effective product differentiation to occur, product consistency is vital. In this respect, dominant food companies have the resources and supply chain control to ensure a large, consistent supply of products that can fulfil specified external or in-house quality standards [33].

Beyond brand equity, dominant food companies invest substantial amounts of money to develop and acquire other types of intangible assets. This includes new product and process innovations (such as inorganic micro-coatings on candy, or the fortification of foods using nano-capsules), brand and packaging designs, and formulas. In some circumstances, these intangible assets can be protected through intellectual property rights channels, conferring a significant long term advantage to the firm [64, 91, 165–169].

Another key barrier to market entry in many processed food markets is the fact that dominant food companies have managed to establish economies of scale in terms of production, marketing and finance. Production economies of scale – achieved through practices such as the acquisition of other firms, large-scale investment in existing production facilities, and investing in advanced process innovations (e.g., automation) – can optimise production efficiency and minimise the marginal cost of production [33, 58, 64, 119, 132, 147, 149, 150, 161–163]. Dominant food companies also make use of marketing economies of scale in which they can lower the costs of marketing practices by spreading their marketing budget across a range of different and effective marketing media [74]. Regarding financial economies of scale, dominant food companies are often able to exploit their ability to access and afford large amounts of cheap capital to make large capital investments [151, 171]. In addition, dominant food companies with subsidiaries in different countries can readily mobilise capital across borders, enabling them to undertake practices such as transfer pricing – namely the trading of goods or services between subsidiaries – in order to minimise tax obligations and maximise financial returns [152, 172, 173]. A core financing strategy of Nestlé, for instance, was described in the literature as locating its trademarks and patents in Switzerland, its home jurisdiction, in order to set up transnational intra-firm royalty payment streams designed to assist the repatriation of profits in tax-effective ways [172].

The ability of dominant food companies to control the processed food supply chain (i.e., ‘supply chain control’) is another important barrier to market entry. For instance, it was identified that dominant food companies often leverage their market power vis-à-vis retailers in order to control distribution channels, including retail shelf space in supermarkets. An example of a related trading practice used by dominant food companies is exclusive dealing arrangements, which entails the placement of restrictions on how much rival product the retailers/distributors are allowed to sell [29, 99, 123, 153, 174, 177, 181, 185, 186, 188]. In some contexts, dominant firms can also aim to be given ‘category captaincy’, which refers to the situation when retailers give food manufacturers leading category management roles, allowing them to arrange and control retail shelf space [118, 178, 182, 184, 187]. Similarly, dominant food companies can make payments for optimal retail positions, known as slotting fees, as well as undertaking calendar marketing agreements, which are payments for preferential treatment by retailers, such as exclusive in-store advertising, during a specified period of time [58, 101, 175, 176, 179, 180, 183]. As a case in point, the European Commission made the decision to address certain trading practices used by TCCC to control distribution channels in an anti-competitive manner, which included forcing a number of distributors to deny access to TCCC’s rivals and using financial incentives to reserve a certain part of retail shelf space dedicated to carbonated soft drinks [181, 242]. Dominant food companies, compared with food manufacturers with less market power, were also described as being able to negotiate more advantageous in-store promotional arrangements with retailers [127].

Given their integration into and access to input markets in different markets around the world, dominant food companies have the ability to pursue supply diversification and competitive sourcing as a means of securing cheaper supplies [82, 116, 132, 162, 189–192]. Additionally, dominant food companies often take advantage of advanced supply chain management practices – such as the use of enterprise resource planning software, and automation innovations in quality management and production – to minimise transaction costs, increase production efficiencies, and ensure product quality and differentiation. Dominant food companies were seen to also acquire or control firms in ancillary industries, such as those involved with storage and refrigeration [82, 147, 155, 193–198]. TCCC, for example, deployed this strategy in its acquisition of a firm involved in the production and distribution of refrigeration units in the European Common Market [199]. Finally, an important supply chain control strategy that can raise barriers to market entry is the ability to obtain, control, and manipulate consumer, market and supply chain-related information. A key means to achieve this was reported as being the use of costly Big Data platform technologies that mine, store and interpret vast amounts of consumer data [132, 201].

### Counter the threat of market disruptors and drive dietary displacement in favour of their products

The review identified three strategies used by dominant food companies to counter the threat of substitute food products: *product diversification*; *control market disruptors*; and *drive dietary displacement and adaption in favour of branded processed foods over alternatives*. The two main groups of substitute products relative to the food products made by dominant food companies were newly developed food products, especially those developed in response to changing consumer trends (e.g. plant-based protein meat and dairy product alternatives), and foods that form part of traditional diets, many of which are unprocessed or minimally processed.

In many cases, the branded products of dominant food companies are also in competition with private label food products across many processed food markets. Private label (eg. supermarket ‘own-brand’) food products are those that are manufactured by a third-party or contract food manufacturer and sold under a retailer’s name. Given that private label products in the same market are technically rival products (that typically compete on price), rather than substitute products per se, we did not include strategies deployed by dominant food companies to counter the threat of private label companies under this strategy as they are addressed as part of other strategies. For example, several studies discussed that dominant food companies with strong brands are likely to be at a lower risk of losing market share to private labels compared with small food companies with lesser known brands [64, 141, 143–145]. Furthermore, in certain contexts, dominant food companies may choose to become a third-party manufacturer for a retailer as part of risk-spreading arrangement (a practice that falls under the *horizontal collaboration* strategy) [243].

A range of product diversification practices were identified during the review, including investing large amounts of money into developing new products [32, 206, 209]; in acquiring firms in substitute markets (e.g. a soft drink manufacturer acquiring a bottled water manufacturer); or by acquiring new brands, especially those linked with being ‘natural’, ‘healthy’, ‘sustainable’, or ‘organic’ [29, 32, 57, 58, 63, 74, 79, 88, 89, 107, 109, 156, 159, 164, 167, 171, 202–206]. Similarly, dominant food companies can also enter substitute markets via strategic alliances or joint ventures, either with large rivals or emerging firms [74, 109, 113, 210, 211].

In order to mitigate the risk of market disruptions, a number of dominant food companies were seen to operate as venture capitalists and company incubators, especially in relation to start-up food technology firms [109, 206, 229]. Indeed, a large number of new firms entering processed food markets are funded by the in-house venture capital programs of dominant food companies, e.g., Nestle’s Terra Accelerator and The Unilever Foundry [109, 206]. The financing role means the large firms are then able to cherry pick and internalise the most successful start-up firms.

Dominant food companies pursue a number of strategies in order to drive dietary displacement away from traditional foods towards their branded processed food products. In addition to increasing consumer demand for their products via intense and aggressive marketing (*refer to section 3.1*), dominant food companies invest in marketing practices to drive changes in consumption habits, such as the promotion of snacking over regular meal times [85]. Dominant food companies also invest substantial amounts of money into engineering hyperpalatable, quasi-addictive, and aesthetically and texturally-pleasing food products [2, 34, 85, 129, 154, 212–214]. For example, a key purpose of their research and development programs is to identify ‘bliss points’ for certain food components (e.g., sugar, salt, fat, and certain additives) in their products in order to optimise consumer pleasure [212–214]. Finally, dominant food companies can take advantage of their production economies of scale, as well as their ability to access cheap commodity inputs, to keep the consumer price of their branded processed food products below that of alternative products [2, 3, 91, 129]. This strategy is particularly important in food product markets in which price plays a key role in the purchasing decisions of consumers.

### Increase firm buyer power by exercising power over upstream actors

It was seen during the review that dominant food companies exercise power over upstream actors in the food supply chain using three related strategies: *integration*, *control* and *coordination*. Dominant food companies vertically integrate ‘backwards’ by acquiring upstream firms, including food production firms and primary food processors [58, 63, 107, 132, 195, 215, 217]. ‘Backwards’ vertical control can be achieved via the imposition of contract agreements [29, 58, 102, 189, 223–226]. In some cases, contract agreements are designed to allow dominant buyers to impose unfair conditions on their suppliers, such as permitting the buyer to make unilateral or retroactive changes to the contract [224].

Dominant food companies were also revealed to use private standards relating to food quality, safety or sustainability as a means of exerting power over their suppliers [29, 132, 190, 221, 222]. Private standards, referring to standards imposed by one private actor on another without the involvement of government, can at times have detrimental effects on small-scale producers. As an example, private standards enforced by Nestle and Parmalat relating to the handling and storage of milk forced thousands of dairy farmers in Brazil out of business, largely because they could neither afford nor access the necessary capital and technology to fulfil the newly imposed private standards [132]. In addition, dominant food companies were revealed to control access to and use of key inputs, such as land, seeds of certain crop varieties, farming equipment, and water [29, 33, 58, 64, 141, 195, 217–220]. Lastly, the coordination of upstream processes and activities can be achieved through the use of risk-spreading arrangements with upstream firms [64, 82, 108, 227–230].

### Increase firm seller power by leveraging power over food retailers and distributors

The review identified that the key strategic approaches for dominant food companies to increase their selling power vis-à-vis downstream actors – that is, the distributors and retailers of their branded products – involved *vertical integration*, *control*, *coordination*, and *collusion*. ‘Forward’ vertical integration is achieved when dominant food companies acquire a retail or distribution firm, or the distribution-related assets of smaller distributors (e.g., dispensing equipment, fridges, and vending machines) [107, 153, 187, 195, 198, 215, 231]. Vertical control over downstream actors can be achieved via a number of trading practices. Some of these trading practices clearly exploit power imbalances and are thus considered unfair to the buyer. Important examples include the use of exclusive dealing arrangements and loyalty rebates to control what the upstream actors can sell [99, 174, 181]; the use of ‘product bundling’ (sometimes referred to as tying) to force buyers to purchase an unwanted product [244]; the use of resale price maintenance, which occurs when manufacturers control the retail price [99]; and the use of territorial supply contracts, in which restrictions are placed on cross-border sourcing, preventing the upstream actor to source the same goods from other national markets where they are cheaper [99, 232].

In a similar fashion to both horizontal and backwards vertical coordination, forward vertical coordination occurs when dominant food companies partake in risk-spreading arrangements with upstream actors in order to better coordinate the distribution of their branded products [206, 228, 233]. Finally, the review identified evidence of dominant food companies colluding with upstream actors by entering into price-fixing arrangements for their branded processed food products [63, 103]. A notable example was the branded bread price-fixing scheme that occurred in Canada between 2001 and 2016 in which colluding firms agreed to coordinate price increases on at least 15 occasions [103].

### Leverage informational power asymmetries in their relations with consumers

The review identified a number of important ways that dominant food companies leverage information-based power asymmetries over consumers. Although it could be justified that all forms of intense and aggressive marketing communications are an expression of power by a firm over a consumer, we primarily focused on strategies and practices described as being exploitative and misleading in nature primarily from a consumer law perspective.

It was revealed that dominant food companies often use marketing practices that specifically target vulnerable population groups [13–16, 234]. As a pertinent example, dominant food companies were reported to have used sophisticated integrated marketing communication practices – combining communication practices such as marketing, advertising, promotions, and public relations – to target young children who do not have the cognitive ability to understand and evaluate marketing strategies [14, 15]. Moreover, on occasions, dominant food companies were reported to bombard children through the simultaneous use of multiple marketing channels, such as TV ads, in-school marketing, product placements in popular programs and movies, and online platforms [15, 16]. The use of online platforms can be particularly exploitative given that large firms are able to collect and use extensive amounts of personal data from young internet users to deliver behavioural-based precision marketing [11]. Another vulnerable population group that dominant food companies were shown to target, predominately through outdoor advertising practices, were people living in lower income neighbourhoods [13, 234].

The review identified a number of cases in which dominant food companies were shown to have taken advantage of informational asymmetries over consumers in relation to food labelling, composition and production processes. Such information based power asymmetries are of notable concern given that product and process-related information cannot easily be found or verified by consumers upon consumption [33, 235, 236]. A number of practices were identified that were considered misleading under consumer law. For instance, the use of misleading representations was reported, including the use of the phrase ‘school canteen approved’ [236]. The use of misleading health claims was also observed, such as Heinz’s claim in the Australian market that one of their products was healthy and nutritious for children, a claim that was subsequently proven to be inaccurate [237]. The alleged use of misleading food composition was also identified in a case in which a dominant food company’s ‘pomegranate-blueberry’ juice beverage was found to contain more than 99% apple and grape juice [235]. Furthermore, it was reported that dominant food companies often use a range of marketing practices on packaging in order to influence consumer purchasing behaviour, such as the use of words with no legal or formal meaning (e.g. natural), the use of unfinished claims (e.g., ‘25% less added salt’ without stating a comparator), irrelevant claims, the use of healthy sounding brand names (e.g. ‘Go Natural’), and the use of ‘greenwashing’ labels (i.e., the practice of marketing a product as being ethical and ecologically friendly without it truly being so) [140, 238].

### Key cross-cutting practices used to achieve multiple strategic objectives

A number of key cross-cutting practices were identified that can be deployed by dominant food companies to achieve multiple strategic objectives. First, the acquisition of other companies or their assets can fulfil different strategic objectives depending on the type of transaction (e.g., horizontal, vertical) and its geographical boundaries (i.e. domestic, transnational). Second, and in a similar manner to firm acquisition, partaking in risk-spreading arrangements (e.g., joint ventures) with other firms can be used to achieve a number of strategic objectives. Third, investing substantial amounts of money into integrated marketing communications can be used to increase consumer demand relative to rivals, drive dietary displacement away from alternative food products, and raise barriers to market entry. Fourth, investing substantial amounts of money into product and process-related research and development and where possible, protecting developed assets via through intellectual property channels, can reduce rivalry by creating new market segments and heighten barriers to market entry. Fifth, collusion can achieve different strategic objectives depending on the parties involved in the arrangement. Finally, the control and manipulation of consumer and supply chain related data can be used to fulfil different strategic objectives depending on which market-based actors are exploited by the resulting informational asymmetries.

## Discussion

The paper identified a number of market strategies and related practices used by dominant food companies to consolidate their power, and categorised these strategies according to six interconnected strategic objectives. Effectively, these six strategic objectives outline how market strategies can be deployed by dominant food companies to leverage power asymmetries within the market environment, especially over consumers, small-scale food producers, smaller manufacturing rivals, and small-scale retailers and distributors. The strategies were identified from a diverse range of literature, including public health, social sciences, competition law and policy, and business and strategic management. The evidence quality and risk of bias of the data sourced from the identified documents were assessed using a novel approach, in which data collected from peer-reviewed articles, court case proceedings, and authoritative reports from inter-governmental organisations were considered to be of higher quality than data sourced from online media content, market research reports, and working papers.

### Public health implications of market strategies used by dominant food companies

There are three broad and interconnected manifestations of public health concern that result from market-based power imbalances that favour dominant food companies: i) the ability to generate profits from anti-competitive behaviour that can then be used to fund corporate practices that undermine public health; ii) the ability to shape retail food environments in ways that promote consumption of unhealthy foods and beverages; and iii) the ability to structure food supply chains that are geared towards the production of cheap inputs used in the manufacturing of unhealthy processed food products. Critically, these public health concerns do not rely on an assumption that smaller, less powerful companies are likely to be more public health conscious. Instead, a central argument for limiting market-power in this area is that smaller, less powerful companies are likely to lack the material resources, capacity and co-ordination required to undermine public health in such a systematic way.

First, and fundamentally, dominant food companies can leverage their market power vis-à-vis all market-based actors to generate and accumulate profits in excess of what would be possible in competitive market environments. The implications of this, besides the broader issues of misallocation and maldistribution of resources, is that dominant food companies are able to accumulate excessive material capabilities, which in turn can be used to fund costly corporate practices that have the potential to undermine public health efforts to improve population diet quality. Some of these corporate practices have non-market dimensions, e.g., lobbying, public relations, investing in scientific research and front groups, and donating to political candidates and campaigns, which serve to influence the interconnected policy, regulatory, institutional, ideological and broader socio-political structures that shape market environments [19]. Other corporate practices that are often funded, at least in part, by profits made from anti-competitive behaviour include a number of market-based practices analysed in this paper. Notably, large food companies devote a substantial amount of resources to create and disseminate intense marketing and related communication strategies that fundamentally serve to maintain and increase consumer demand [2, 85, 245]. As a case in point, the advertising budget of The Coca-Cola Company alone in 2019 (approximately USD 4.25 billion) was nearly the same as the entire 2018–2019 programme budget of the World Health Organization (approximately USD 4.42 billion) [246, 247]. In many cases, these marketing and communication practices are carefully designed to manipulate and distort consumer judgement [248]. Furthermore, dominant food companies have the ability to rapidly adapt their marketing and communication strategies to exploit events and dynamics that take place in the broader socio-political environment (e.g., Black Lives Matter movement, COVID-19 pandemic) [249–251].

A second important public health implication of the market power of dominant processed food manufacturers is that it increases their ability to directly shape retail food environments – in some cases via the use of anticompetitive practices, such as exclusive dealing arrangements and slotting fees. There is widespread recognition that unhealthy food environments, in which unhealthy food products are widely available and heavily promoted, are a major driver of unhealthy diets and obesity globally [5, 252]. Accordingly, where market-based power imbalances favour companies that generate profits from the sale of unhealthy food and beverages, it is likely to negatively affect population diets and related health outcomes.

Third, the market power of dominant processed food manufacturers over small-scale producers can promote and reinforce the production of unhealthy processed food products, particularly in cases where the product portfolios of dominant manufacturers are mainly comprised of unhealthy food products. In order to remain economically viable, small-scale producers often have little choice but to produce commodity crops for dominant processed food manufacturers, instead of engaging in diverse crop production for healthier and more sustainable local food systems [4, 53].

There are also a number of manifestations of market power in which the public health implications are less clear. For example, the entry and penetration of ‘healthier’ substitute markets by dominant food companies, largely in response to consumer concerns and trends, could, on the surface, be perceived as beneficial for public health. The drive to differentiate and diversify products, for instance, based on issues of sustainability (e.g., shifting towards plant-based proteins) and health (e.g., promotion of fortified, functional and formulated food products) could lead to some public health benefits [18, 29, 253]. However, the public health community must remain vigilant in exploring whether these potential benefits are outweighed by more fundamental issues, such as how these strategies might lead to further consolidation of power in the hands of dominant food companies. Proponents of corporate technology-orientated solutions to improve population diet quality often contend that consolidated corporate power is not a concern in and of itself, given that powerful transnational firms are best placed to mobilise the necessary resources [4, 18, 53, 254]. Critics of this view, however, have pointed out that such a way of thinking is reductionist and could lead key policy makers to favour policy solutions that maintain the power of manufacturers of unhealthy foods at the expense of policy solutions that more comprehensively reform the healthiness of food systems [4, 53, 156, 254–256].

### External factors that shape (and are shaped by) market strategy

Although the main context of analysis of this paper was the market environment, it is important to highlight that interconnected factors ‘external’ to the market environment – i.e. the broader economic, political-legal, socio-cultural, demographic, technological, and ecological systems and processes – shape, and are shaped by, the market strategies of dominant food companies [21, 24, 257]. In this respect, market strategy analysis should not be a stand-alone inquiry, but instead serve to complement both non-market corporate strategy analyses and the examination of how underlying systemic processes, dynamics and paradigms (e.g. globalisation, liberalisation, financialisation) can facilitate and promote corporate interests over those of public health [6].

Market structures and market strategies are heavily shaped by underlying global and jurisdictional political economies. For instance, the market strategies of dominant food companies are influenced by trade and investment policy [116, 258, 259], competition policy [100], food and agricultural policy [113, 260], and fiscal and financial policy [261]. Such policies, laws, regulations and institutions vary significantly across jurisdictions. Market strategies, for instance, are less likely to be regulated in liberal market economies, in countries with weak regulatory institutional arrangements, or if they are transnational in nature and operate transboundary across and within states [262, 263]. Broader economic structures and processes also play a key role in shaping the market strategies of dominant food companies. For example, globalising economic processes, such as the integration of trade, investment, information and knowledge flows into global value chains, have enabled and promoted the emergence of enormous transnational food corporations [29, 80, 116]. Furthermore, the economic development that has occurred in many countries in recent decades has increased the purchasing power of a greater number of consumers, in turn boosting the sales growth of branded processed food products [2, 264]. Conversely, consumer confidence and the sales of branded processed food products have tended to drop during macro-economic shocks, such as those associated with financial crises, which often lead to currency devaluation, increased unemployment and inflation [86, 265].

A range of socio-cultural and demographic drivers have facilitated population-level dietary shifts from freshly prepared meals to ready-to-consume, processed food products, notably urbanisation, the rise in paid employment for women and their engagement in less traditional gender-based roles, irregular working hours, a desire for cheap and convenient meals, reduced family size, and an increase in one-person households [91, 126, 170, 205]. Dominant food companies have also been able to harness ideas and values linked with modernisation and social desirability to aggressively market their branded food products, especially in emerging markets [91].

Market strategy is also shaped by technology-related innovations. Pertinent examples specific to dominant food companies include scientific developments in food processing and nutrition research (e.g. nanotechnology and biotechnology) [18, 108, 167, 209]; technological innovations in transportation and storage (e.g. just-in-time transportation technologies, cold chain technology) [132]; improvements in communication technology [91, 108]; Big Data platform technologies (especially with regard to targeted and integrated marketing) [201, 266]; and food production-related technological innovations, such as those that increase and standardise commodity production [33, 267].

### Policy implications and actions to address market-based power imbalances

One of the goals of market strategy analysis of dominant food companies from a public health perspective is to identify policy actions that have the potential to address the market-based power imbalances that drive unhealthy diets. It is important to note that, while the focus of this paper is on policies designed to protect and promote public health, there are clear and important interconnections between public health goals and broader social and ecological goals.

Competition policy, at least in principle, plays an important role in regulating food supply chains by approaching and enforcing issues such as merger control, unfair trading practices, and vertical abuse of power [99, 153, 263]. Horizontal, vertical and cross-border merger control can prevent transactions that would further consolidate the market power of dominant food companies, as well as regulate their entry and penetration into emerging markets [63, 153, 263, 268, 269]. Additionally, the strengthened enforcement of unfair trading practices and vertical abuses, such as the use of slotting fees and ‘category captaincy’, could play an important role in promoting healthier food environments by reducing the ability of dominant food companies to control retail shelf space [175, 178, 179].

However, it has often been argued that most competition policy regimes around the world have limited scope to promote healthier food systems [270, 271]. One fundamental reason for this is the narrow economic approach used to interpret consumer welfare in competition-related decision making. Notably, consumer price (and, to a lesser extent, availability and innovation) typically trumps broader social and ecological concerns, such as the healthfulness of food environments, in consumer welfare assessments [269, 271, 272]. This narrow economic approach to consumer welfare promotes the idea that cheap, widely available and unhealthy food products are beneficial, and not detrimental, to consumer welfare [272, 273].

One approach to reforming competition policy that has the potential to benefit public health could be to integrate the right to food, and in particular the right to adequate food, into the interpretation and application of competition policy [272–274]. Such an approach could ensure that competition authorities facing a choice between different interpretations of competition law involving dominant food companies (e.g., during assessments of proposed mergers) would be required to select the option that best protects and promotes public health whilst preserving the process of competition [273]. Similarly, broadening the scope of competition policy could help to counter industry group opposition to the implementation of certain public health policies, such as health-related taxes, on the grounds that they would distort competition [261, 272, 275–277]. There are important legal precedents in which the social benefits of implementing health-related taxes have been considered to outweigh the social costs of the potential distortion of competition. In 2018, for instance, the European Commission decided that the scope of the sugar-sweetened drinks tax implemented in Ireland was consistent with the health and nutrition objectives pursued by the government, and would not distort competition in an unduly manner [278].

Curbing the power of dominant food companies, as part of efforts to build healthier food supply chains, could also be partly achieved through policy action that supports food producers to participate in local and diversified food production, instead of being locked into commodity crop production. For instance, governments across different levels of governance could invest in infrastructure supporting local food supply chains for perishable products (e.g. improved transport and cold storage infrastructure); supporting farmers to engage in direct sales (e.g. participation in produce markets) and local processing and value-adding activities (e.g. food hubs); the upgrading of health, food quality and phytosanitary legislation to help small producers overcome existing restraints; increasing support for alternative food business models (e.g. food cooperatives); developing integrated agricultural knowledge and innovation systems that could help to address information asymmetries; and public procurement policies that mandate the procurement of healthy, local and sustainable food products [269, 279, 280].

Policy actions that support vulnerable consumers overcome information-based power imbalances are also likely to play an important role in promoting healthy diets. For instance, countering exploitative marketing practices could be partly achieved via the prohibition of marketing, including digital marketing, of unhealthy foods to children, and the ban of unhealthy food marketing in school areas, on low shelves or checkout counters in supermarkets, and in TV programs scheduled at times when children typically watch TV. Packaging-related information asymmetries could also be partly overcome by establishing mandatory front-of-pack labelling schemes [269, 281, 282]. Finally, vulnerable consumers could be supported through policy actions that focus on health and diet quality equity. Such actions include those designed to improve the affordability and accessibility of healthy food alternatives, such as tax exemptions and subsidies on fresh produce and taxation of unhealthy products, urban policy that focuses on the distribution of healthy food options in deprived and vulnerable urban neighbourhoods, and greater access to fresh drinking water [269, 279, 283].

### Contribution to the public health literature

The typological framework presented in this paper is well-placed to inform a comprehensive examination of the market strategies and related practices used by dominant food companies within and across jurisdictions. Such studies will require the development of specific protocols to guide analyses. One example of a future research avenue could be to examine the market strategies used by a specific company, such as The Coca-Cola Company, across multiple countries over an extended period of time. Another potential research avenue could be to identify the aggregated market strategies used by the dominant market players of a specific food industry, such as sugar-sweetened beverages, within a specific jurisdiction. In both cases, research could include an examination of the use (or lack thereof) of remedies implemented by the relevant national competition authorities in response to the deployed market strategies.

More broadly, the presented market strategy framework can assist public health efforts to understand and address the ways in which corporate strategies influence health. To date, the majority of corporate strategy frameworks within the public health literature have largely focused on the non-market dimensions of strategies deployed by firms active in health-harming industries [9, 284–287]. This emphasis on non-market strategy is well targeted, particularly in highly regulated industries such as tobacco, wherein firms have substantial incentives to secure favourable policy and regulatory environments to the detriment of public health [288]. Yet, firms in health-harming industries deploy both market and non-market strategies to consolidate power and maximise profits. Although a number of studies in the field of public health have, collectively, examined a variety of market strategies used by firms across a number of health-harming industries [245, 289–295], the approach to examine market strategies has not been systematic in the same way it has been for non-market strategies. In this respect, the presented typological framework could be used to complement existing political strategy analyses of dominant food companies [296–299], thereby broadening understanding of the wide range of corporate strategies used by dominant food companies that influence health and diet. Furthermore, this paper could serve as a blueprint for future public health research to systematically review the market strategies used by corporations in other food sectors (such as retail and food service), as well as corporations in other health-harming industries.

### Strengths and weaknesses of the study

A key strength of this paper is that it is a systematic review covering a range of literature – notably public health, business, strategic management, and competition policy – that explores market strategy from a number of different perspectives. Furthermore, we used a novel approach to assess evidence quality and risk of bias. Another key strength of this paper is that it uses Porter’s Five Forces framework to inform market strategy analysis, a framework that is very well-established throughout the business and strategic management literature and is well-placed to explore market-based power asymmetries. Although the use of Porter’s Five Forces in the public health literature is not novel, to the best of our knowledge this is the first public health paper to use Porter’s Five Forces framework to systematically analyse the use of market strategies used by corporation active in any health-harming industry or sector.

The paper has a number of important limitations. First, given the nature and subjectivity of interpreting power, there was an inevitable element of subjectivity in the way we framed different strategies according to the six strategic objectives. Second, there are clear interconnections and crossovers with a number of the strategies discussed, particularly in light of the difficulty in accurately delineating and defining food product markets and market segments. For example, there is a clear overlap between market segmentation, product differentiation and product diversification strategies, as well as the strategies linked with increasing consumer demand and building brand value. Third, it is important to note that certain sectors of the food industry may have unique market characteristics that cannot be generalised to the food manufacturing sector more broadly. For instance, breast milk substitute manufacturers may attempt to control distribution channels, such as health care and pharmaceutical channels, that are not generally used by firms active in other sectors of the food industry. Finally, this paper focused on dominant firms considered to primarily be processed food manufacturers. It is important to note that dominant companies from other functional levels have also integrated into the food manufacturing sector. The market dominance of actors in other sectors – such as powerful retailers (e.g., large supermarket chains), commodity traders (e.g., Archer Daniels Midland) and meat or dairy processors and wholesalers (e.g. WH Group, Fonterra) – can confer specific competitive advantages not covered in this paper. Given this limitation, a potential avenue for future research could be to look at the market-based power asymmetries specific to the different functional levels of the food value chain.

## Conclusion

This paper has presented a novel typological market strategy framework specific to dominant food companies and their use and consolidation of market power vis-à-vis other market-based actors. Informed by Porter’s Five Forces framework, the presented framework outlines key market strategies and related practices in relation to six key strategic objectives. The presented framework is well-placed to inform analysis of market strategies used by dominant food companies as part of public health efforts to understand and address the ways in which the use of corporate strategies by dominant food companies can undermine public health and diet quality. In addition, the presented market strategy framework is well-placed to identify leverage points within a specific jurisdiction that could be targeted by policy actions to address market-based power imbalances. Notably, competition policies related to merger control and the misuse of market power have the potential to create healthier food systems. Given that in many cases competition authorities fail to incorporate the necessary range of social and ecological considerations that concern societal welfare, the public health community should aim to position itself as an important driver of competition policy reform that truly takes consumer welfare and public interest considerations into account.

## Supplementary Information

**Additional file 1.**

**Additional file 2.**

## Data Availability

All data generated or analysed during this study are included in this published article [and its supplementary information files].
