# Supplementary material for: Market strategies used by processed food manufacturers to increase and consolidate their power: a systematic review and document analysis
Source: Global Health. 2021 Jan 26;17:17. doi: 10.1186/s12992-021-00667-7 (PMC7836045; doi:10.1186/s12992-021-00667-7)
Supplement: Supplementary file 1 — Additional file 1. [file 12992_2021_667_MOESM1_ESM.docx]

**Search terms**

Public health, social sciences, business and strategic management literature were searched via Scopus, Web of Science, Medline, Business Source Complete, and ABI Inform using the string:

*(food OR beverage OR drink) AND (manufactur! Or process!) AND (‘market strateg!’ OR ‘business strateg!’ OR ‘corporate strateg!’ OR ‘business tactic*’ OR ‘business practice*’) AND (‘market power’ OR ‘corporate power’ OR ‘bargaining power’ OR ‘purchas! power’ OR ‘monopoly power’ OR ‘monopsony power’ OR ‘sell! power’ OR ‘buy! power’)*

Competition law and policy literature and online news articles were searched via Thomas Reuters Westlaw, Lexis Advance, Factiva and NewsBank using the string:

*(food OR beverage OR drink) AND (manufactur! Or process!) AND (‘market strateg!’ OR ‘business strateg!’ OR ‘corporate strateg!’) AND ‘market power’*
