# Supplementary material for: Market strategies used by processed food manufacturers to increase and consolidate their power: a systematic review and document analysis
Source: Global Health. 2021 Jan 26;17:17. doi: 10.1186/s12992-021-00667-7 (PMC7836045; doi:10.1186/s12992-021-00667-7)
Supplement: Supplementary file 2 — Additional file 2. [file 12992_2021_667_MOESM2_ESM.doc]

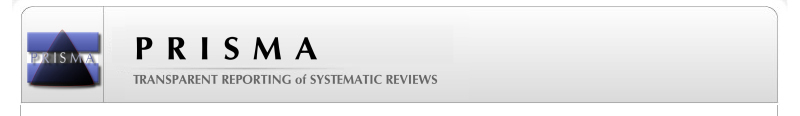
**PRISMA Flow Diagram**

**Screening**

**Included**

**Eligibility**

**Identification**

Records identified through database searching
n = 1115

Additional records identified through other sources
n = 103

Records after duplicates removed
n = 1140

Records screened
n = 1140

Records excluded
n = 849

Full-text articles assessed for eligibility
n = 291

Full-text articles excluded
n = 78

Studies included in qualitative synthesis
n = 213
